# Impact of the cost-of-living crisis on patient preferences towards virtual consultations

**DOI:** 10.1177/1357633X241255411

**Published:** 2024-05-20

**Authors:** Tetiana Lunova, Katherine-Helen Hurndall, Roberto Crespo, Peter Howitt, Melanie Leis, Kate Grailey, Ara Darzi, Ana L Neves

**Affiliations:** 1 NIHR North West London Patient Safety Research Collaboration (PSRC), Institute of Global Health Innovation, 4615 Imperial College London, London, UK; 2 NIHR Applied Research Collaboration (ARC) NWL, 4615 Imperial College London, London, UK; 3 Center for Health Policy, Institute of Global Health Innovation, Imperial College London, London, UK; 4 Department of Primary Care and Public Health, 4615 Imperial College London, London, UK

**Keywords:** Virtual consultations, cost-of-living crisis, virtual care, patient preferences, teleconsulting, telehealth‌

## Abstract

**Introduction:**

Since 2021, the world has been facing a cost-of-living crisis which has negatively affected population health. Meanwhile, little is known about its impact on patients’ preferences to access care. We aimed to analyse public preference for the modality of consultation (virtual vs face-to-face) before and after the onset of crisis and factors associated with these preferences.

**Methods:**

An online cross-sectional survey was administered to the public in the United Kingdom, Germany, Italy and Sweden. McNemar tests were conducted to analyse pre- and post-crisis differences in preferences; logistic regression was used to examine the demographic factors associated with public preferences.

**Results:**

Since the onset of crisis, the number of people choosing virtual consultations has increased in the United Kingdom (7.0% vs 9.5% *P* < 0.001), Germany (6.6% vs 8.6%, *P* < 0.008) and Italy (6.0% vs 9.8%, *P* < 0.001). Before the crisis, a stronger preference for virtual consultations was observed in people from urban areas (OR 1.28, 95% CI 1.05–1.56), while increasing age was associated with a lower preference for virtual care (OR 0.966, 95% CI 0.961–0.972). Younger people were more likely to switch to virtual care, while change to face-to-face was associated with younger age and lower income (OR 1.34, 95% CI 1.12–1.62). Older adults were less likely to change preference.

**Conclusions:**

Since the onset of the cost-of-living crisis, public preference for virtual consultations has increased, particularly in younger population. This contrasts with older adults and people with lower-than-average incomes. The rationale behind patients’ preferences should be investigated to ensure patients can access their preferred modality of care.

## Introduction

Over recent years, the world has faced several unprecedented challenges. The COVID-19 pandemic, followed by military destabilisation in Europe, has contributed to a major cost-of-living crisis that resulted in significant price and tax rises, drastic social security cuts, as well as rent and energy bill hikes.^1,2^ As the cost of living has risen significantly and wages have stayed generally the same, people have been forced to make drastic cuts in their daily expenses and adjust their lifestyles accordingly.^3–5^ Evidence suggests that people are choosing less expensive supermarkets, reducing recreational overseas travel and decreased their spending on clothes and leisure activities.^6^ More people are choosing public transport instead of driving a car and eliminating non-essential journeys due to substantial increases in fuel prices.^7,8^ In light of these forced adjustments, people's decisions regarding healthcare could have also changed. The high cost of living may lead individuals to delay seeking medical care due to concerns about associated expenses, travel affordability and busy schedules. By prioritising immediate financial necessities, individuals may become less inclined to invest in preventative healthcare.

The cost-of-living crisis has also widened pre-existing health inequalities. A growing body of evidence indicates that low-income households, older people, and those from ethnic minorities have been disproportionately affected by the crisis, particularly with respect to their physical and mental health.^9^

Virtual consultations demonstrated their benefit during the COVID-19 pandemic, as they allowed patients to receive timely consultations from their doctors without putting themselves at risk of getting infected.^10,11^ At the same time, the growing popularity of virtual care has exposed significant inequalities, with some population groups not being able to benefit from digital technology.^12^

Since the World Health Organization has officially announced the end of the COVID-19 pandemic as a global health emergency, most patients have been provided the option to choose between an in-person appointment with a clinician or seeing the same clinician virtually.^13^ However, virtual consultations remain deeply embedded in everyday medical care.

Considering the magnitude of the cost-of-living crisis's impact on people's health and lifestyle, it can be hypothesised that public preferences for the modality of appointment may have also been affected. Admittedly, the way people wish to receive healthcare depends on a multitude of socio-economic determinants, such as income, social protection and job security, and these have been significantly compromised by the crisis. It can be assumed that some known benefits of virtual consultations, such as time and geographical flexibility could have wide implications for patients in the cost-of-living crisis times as they allow people to avoid travel and parking expenses, taking time off work, or securing childcare.^10^ However, as of now, evidence is scarce on whether the cost-of-living crisis has indeed changed patients’ preferences for the modality of appointment and which patients might have been more likely to change.

This study analysed the public preference for the modality of consultation (i.e. face-to-face or virtual) in four high-income countries before and after the onset of the cost-of-living crisis. As a secondary aim, factors associated with these preferences have been explored.

## Methods

### Study design and setting

This is a cross-sectional study that used an online questionnaire survey of participants in four high-income countries: the United Kingdom, Germany, Italy and Sweden. The study adhered to the Strengthening the Reporting of Observational Studies in Epidemiology (STROBE) guideline for cross-sectional studies.^14^

### Participants

The participants were recruited via YouGov, an international online research data and analytics technology group.^15^ To be eligible for the study, the participants had to be older than 18 years of age and be able to read and write in their resident country's language. To ensure each country's representative samples, YouGov drew a subsample of participants representative of the national average in terms of baseline characteristics. The survey took place between 17 March 2023 and 30 March 2023.

### Questionnaire description

The questionnaire was available in four languages: English in the United Kingdom, German in Germany, Swedish in Sweden and Italian in Italy. The questionnaire was developed by our multidisciplinary team of researchers and contained questions about participants’ baseline characteristics such as age, gender, location (i.e. urban, rural, mixed) and income. The questionnaire also contained questions about ethnicity and region of residence within each country; however, this data was not used in this study due to high heterogeneity. For descriptive purposes, age and income were categorised, respectively, in age groups (18–29, 30–39, 40–49, 50–59, 60–69, over 70 years old) and income terciles (lower, middle and upper) (Table 1). The rationale for grouping the participants into terciles was uneven income distribution in the cohort. In the regression model, age was analysed as a continuous variable. The income stratification was performed on an individual-country basis to account for any inter-country variations. Pre-crisis preferences for healthcare delivery were captured by asking, ‘Which, if either, of the following was your preferred mode of care delivery before the cost-of-living crisis began in late 2021?’ and current preferences were captured by asking, ‘Thinking about currently … Which, if either of the following, is your preferred mode of care delivery?’ with possible answers ‘face-to-face’, ‘virtual’, ‘no preference’, ‘don't know’ (Table 2). The research questions in this study were answered by two questions housed within a more extensive YouGov questionnaire. The questionnaire did not specify the setting of virtual consultations (i.e. primary or secondary care) but referred to respondents’ preferences for this modality in general.

**Table 1.** Categorisation of income into income terciles.

	T1	T2	T3
UK	Under £5000–£19,999 per year	£20,000–£39,999 per year	£40,000 and over
Germany	less than 500 €–2000€ per month	2001€–4000€ per month	4001€ and over
Sweden	less than 100,000 kr–399,999 kr	400,000 kr–799,999 kr	800,000 kr and over
Italy	less than 5.000 €–19,999€ per year	20.000 €–39.999 € per year	39.999 € and over

**Table 2.** The description of YouGov questionnaire.

Question	
Thinking about before the cost-of-living crisis began in late 2021 – Which, if either, of the following was your preferred mode of care delivery?	Face-to-face Virtual No preference Don’t know
And now thinking about currently – Which, if either, of the following is your preferred mode of care delivery?	Face-to-face Virtual No preference Don’t know
Age (years)	18–99
Country	1) UK 2) Sweden 3) Italy 4) Germany
Gender	1. Male 2. Female
Location	1. Urban 2. Mixed 3. Rural
Household income	*Germany* 1) less than 500 € per month 2) 501€ to 1000€ per month 3) 1001€ to 1500€ per month 4) 1501€ bis 2000€ per month 5) 2001€ bis 2500€ per month 6) 2501€ bis 3000€ per month 7) 3001€ bis 3500€ per month 8) 3501€ bis 4000€ per month 9) 4001€ bis 4500€ per month 10) 4501€ bis 5000€ per month 11) 5001€ bis 10,000€ per month 12) 10.001 € and more per month 13) Do not wish to disclose *Sweden* 1) less than 100,000 kr per year 2) 100,000 kr till 199,999 kr per year 3) 200,000 kr till 299,999 kr per year 4) 300,000 kr till 399,999 kr per year 5) 400,000 kr till 499,999 kr per year 6) 500,000 kr till 599,999 kr per year 7) 600,000 kr till 699,999 kr per year 8) 700,000 kr till 799,999 kr per year 9) 800,000 kr till 999,999 kr per year 10) 1,000,000 kr till 1,199,999 kr per year 11) 1,200,000 kr till 1,499,999 kr per year 12) 1,500,000 kr till 1,999,999 kr per year 13) 2,000,000 kr till 2,499,999 kr per year 14) 2,500,000 kr till 3,499,999 kr per year 15) 3,500,000 kr till 4,999,999 kr per year 16) 5,000,000 kr or more per year 17) do not know 18) do not wish to disclose *Italy* 1) less than 5.000 € per year 2) 5.000 € a 9.999 € per year 3) 10.000 € a 14.999 € per year 4) 15.000 € a 19.999 € per year 5) 20.000 € a 24.999 € per year 6) 25.000 € a 29.999 € per year 7) 30.000 € a 34.999 € per year 8) 35.000 € a 39.999 € per year 9) 40.000 € a 44.999 € per year 10) 45.000 € a 49.999 € per year 11) 50.000 € a 59.999 € per year 12) 60.000 € a 69.999 € per year 13) 70.000 € a 99.999 € per year 14) 100.000 € a 149.999 € 15) 150.000 € and more 16) 500,000€ and more 17) Do not know 18) Prefer not to say *UK* 1) under £5000 per year 2) £5000 to £9999 per year 3) £10,000 to £14,999 per year 4) £15,000 to £19,999 per year 5) £20,000 to £24,999 per year 6) £25,000 to £29,999 per year 7) £30,000 to £34,999 per year 8) £35,000 to £39,999 per year 9) £40,000 to £44,999 per year 10) £45,000 to £49,999 per year 11) £50,000 to £59,999 per year 12) £60,000 to £69,999 per year 13) £70,000 to £99,999 per year 14) £100,000 to £149,999 per year 15) £150,000 and over 16) Don't know 17) Prefer not to answer

### Data analysis

Out of all participants who completed the questionnaire (*n* = 8152), only those who stated their current preference for either virtual or face-to-face consultations were included in the study (*n* = 6391, 78.4%). Continuous data were summarised as mean ± standard deviation and categorical data as total and relative frequencies. Differences in the participants’ preferred mode of care delivery before the cost-of-living crisis and current preferences were analysed using the McNemar test. Logistic regression was used to examine the factors associated with participants’ preferences for care delivery (i.e. gender, age, income, location). Factors associated with public preference for virtual versus face-to-face care were first analysed in the multivariate analysis. Only covariates statistically significant in the univariate analysis were included in the multivariate model. The significance level was set at *P*-value (*P*) < 0.05 for all analyses. All calculations were conducted using IBM Statistical Package for the Social Sciences (SPSS) v. 29.0.

### Ethics

Ethical approval was granted by Imperial College London's Ethics Research Committee (Reference number: 6542531).

## Results

A total of 6391 participants were included in the study, among which 1459 (23.0%) participants came from the United Kingdom, 1597 (25.0%) were from Germany, 1723 (27.0%) from Italy and 1612 (25.0%) from Sweden. Most respondents were 30–69 years old, with only 875 (13.7%) younger than 29 and 777 (12.1%) over 70 years old. There was close to equal representation of both genders: 3127 (49.0%) men and 3264 (51.0%) women. According to reported income, most participants were classified into lower and middle-income terciles, 1746 (27.3%) and 2046 (32.0%) respectively, while 1503 (23.5%) participants reported higher-than-average annual income. Most respondents in this study came from urban backgrounds 3956 (61.9%), 1446 (22.6%) came from rural and 931 (14.6%) from mixed backgrounds (Table 3).

**Table 3.** Characteristics of the participants according to their change in care delivery preferences.

Characteristics	Patients’ preferences before the COL crisis			Changes in patients’ preferences after the COL crisis		Total
	Participants who preferred F2F care before crisis	Participants who preferred virtual care before crisis	Participants who did not have any preference	Participants who changed their preference to virtual	Participants who changed their preference to F2F	
*Country*						
The United Kingdom	1226 (22.9%)	127 (25.7%)	106 (19.7%)	106 (23.8%)	121 (18.8%)	1459 (23.0%)
Germany	1319 (24.6%)	117 (23.7%)	161 (30.0%)	101 (22.7%)	194 (30.1%)	1597 (25.0%)
Italy	1519 (28.3%)	101 (20.4%)	103 (19.2%)	137 (30.8%)	133 (20.6%)	1723 (27.0%)
Sweden	1296 (24.2%)	149 (30.2%)	167 (31.1%)	101 (22.7%)	197 (30.5%)	1612 (25.0%)
Missing	0 (0%)	0 (0%)	0 (0%)	0 (0%)	0 (0%)	0 (0%)
*Gender*						
Male	2616 (48.8%)	250 (50.6%)	261 (48.6%)	210 (47.2%)	326 (50.5%)	3127 (49.0%)
Female	2744 (51.2%)	244 (49.4%)	276 (51.4%)	235 (52.8%)	319 (49.5%)	3264 (51.0%)
Missing	0 (0%)	0 (0%)	0 (0%)	0 (0%)	0 (0%)	0 (0%)
*Age*						
18–29	665 (12.4%)	117 (23.7%)	93 (17.3%)	101 (22.7%)	120 (18.6%)	875 (13.7%)
30–39	824 (15.4%)	141 (28.5%)	71 (13.2%)	102 (23%)	103 (16%)	1036 (16.2%)
40–49	925 (17.2%)	91 (18.4%)	91 (17.0%)	81 (18.1%)	109 (16.8%)	1107 (17.3%)
50–59	1108 (20.7%)	73 (14.7%)	104 (19.4%)	88 (19.8%)	115 (17.9%)	1285 (20.1%)
60–69	1164 (21.7%)	46 (9.3%)	101 (19.0%)	51 (11.5%)	115 (17.9%)	1311 (20.5%)
70+	674 (12.6%)	26 (5.4%)	77 (13.3%)	22 (4.9%)	83 (12.8%)	777 (12.1%)
Missing	0 (0%)	0 (0%)	0 (0%)	0 (0%)	0 (0%)	0 (0%)
*Income*						
Lower tercile	1427 (26.6%)	155 (31.4%)	164 (30.5%)	143 (32.1%)	207 (32.1%)	1746 (27.3%)
Middle tercile	1737 (32.4%)	154 (31.2%)	155 (28.9%)	129 (28.9%)	193 (29.9%)	2046 (32.0%)
Higher tercile	1264 (23.5%)	135 (27.3%)	104 (19.4%)	112 (25.2%)	130 (20.1%)	1503 (23.5%)
Don’t know/Prefer not to answer	930 (17.3%)	48 (9.72%)	114 (21.2%)	61 (13.7%)	114 (17.7%)	1092 (17.1%)
Missing	2 (0.1%)	2 (0.4%)	0 (0%)	0 (0%)	1 (0.15%)	4 (0.06%)
*Location*						
Urban	3317 (61.9%)	335 (67.8%)	304 (56.6%)	278 (62.5%)	384 (59.9%)	3956 (61.9%)
Mixed	785 (14.6%)	61 (12.3%)	85 (15.8%)	60 (13.5%)	95 (14.7%)	931 (14.6%)
Rural	1206 (22.5%)	95 (19.2)	145 (27.0%)	105 (23.6%)	162 (25.1%)	1446 (22.6%)
Missing	52 (1.0%)	3 (0.6%)	3 (0.6%)	2 (0.4%)	4 (0.6%)	58 (0.9%)

F2F: face-to-face appointments.

### Impact of cost-of-living crisis on public's preference towards virtual consultations

**Figure 1.**
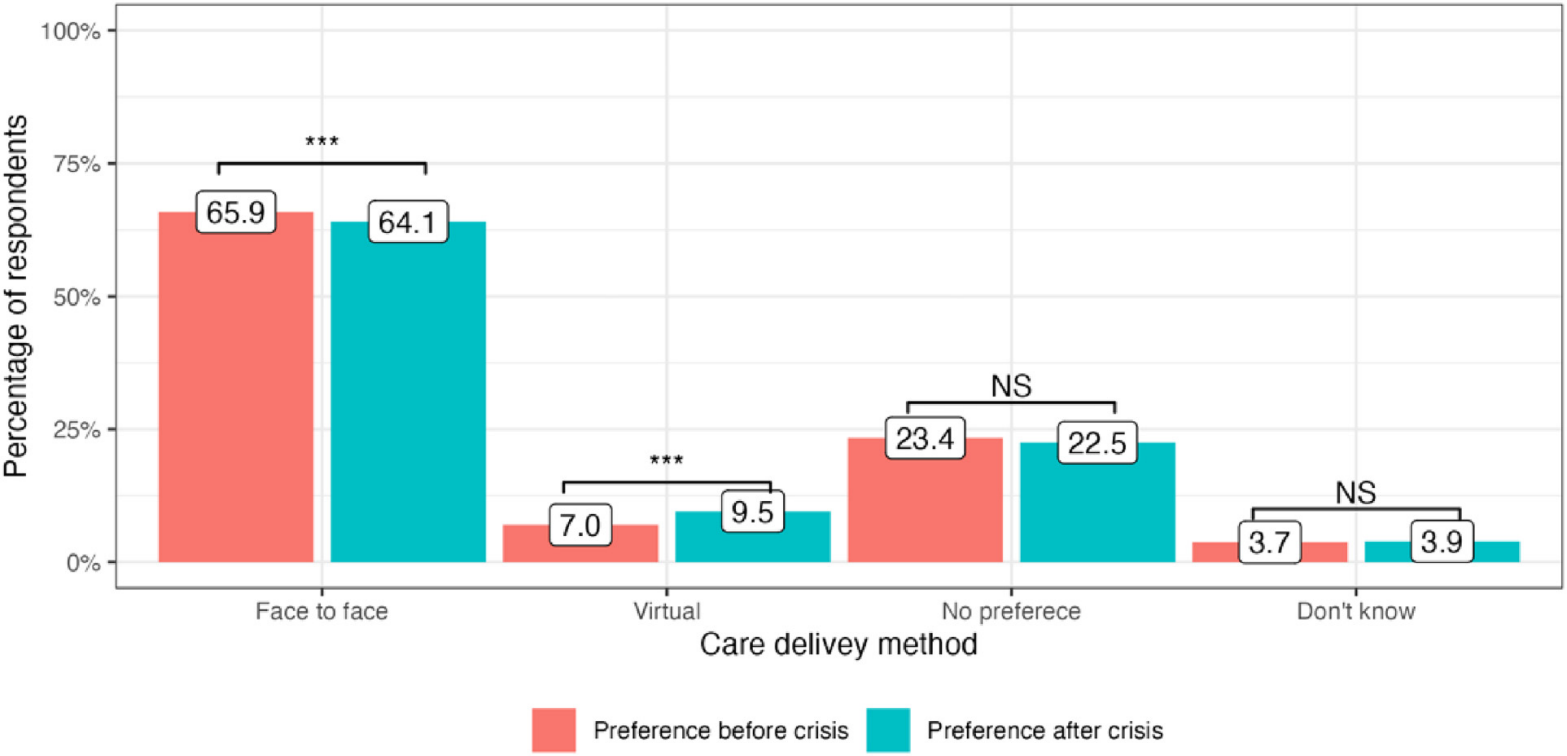
Public preference for healthcare delivery in the United Kingdom.

**Figure 2.**
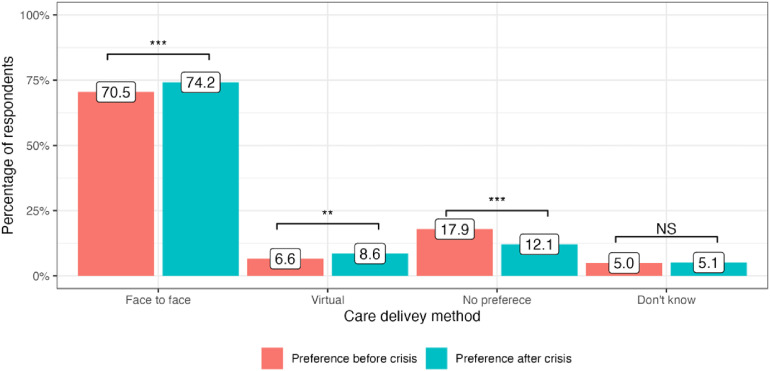
Public preference for healthcare delivery in Germany.

**Figure 3.**
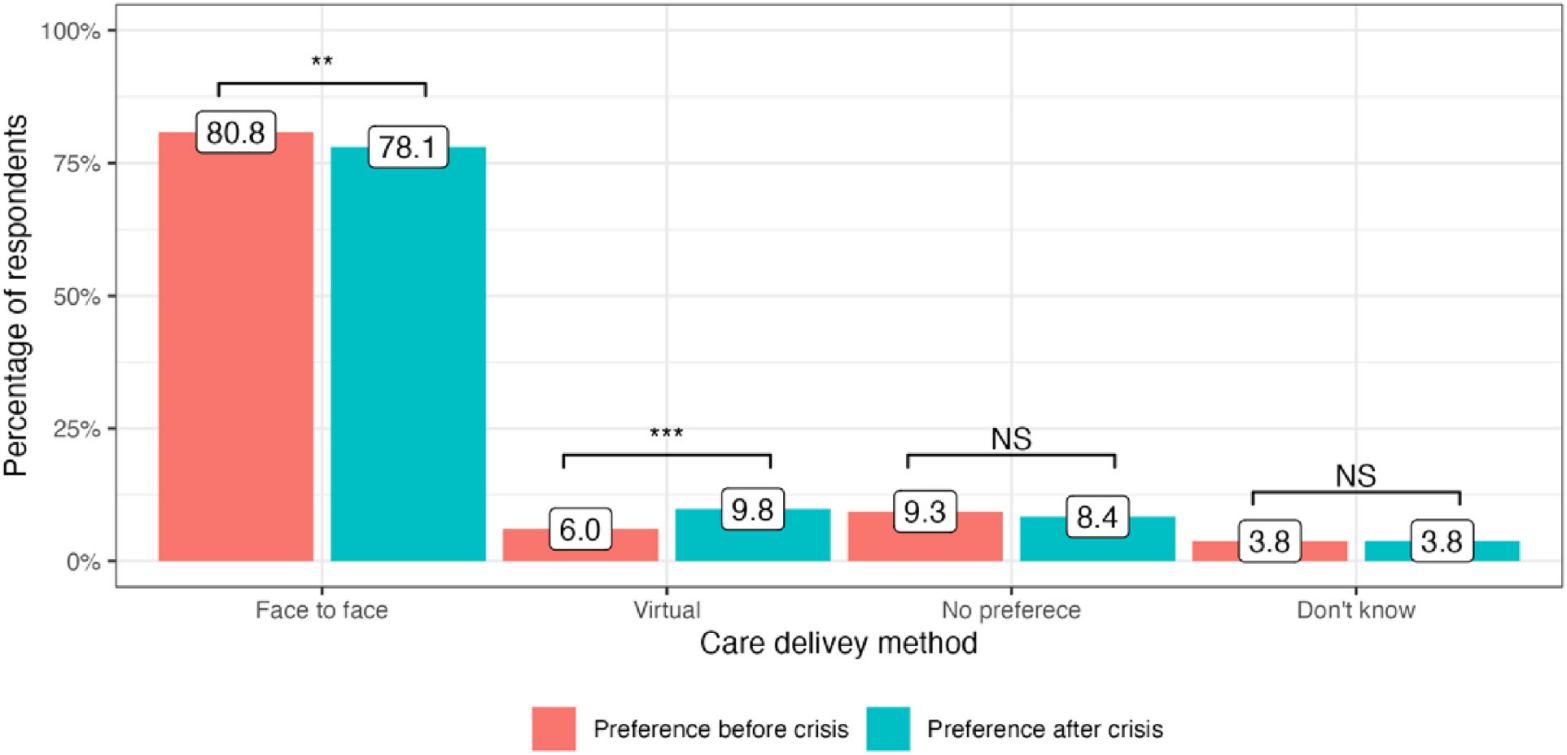
Public preference for healthcare delivery in Italy.

**Figure 4.**
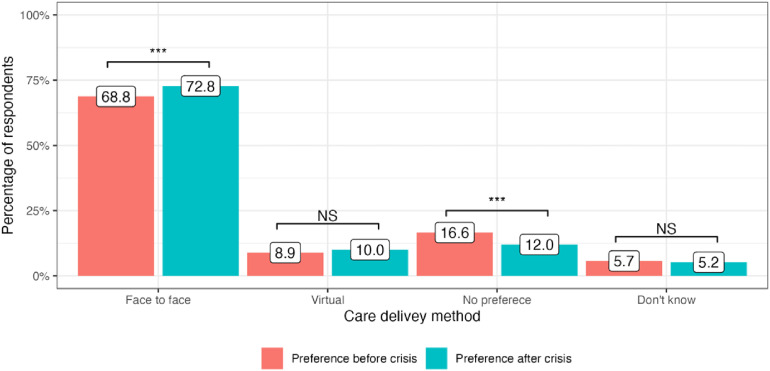
Public preference for healthcare delivery in Sweden.

The cost-of-living crisis has driven significant changes in public preferences for the delivery of medical services in all four included countries. In the United Kingdom, there has been a decrease in preference for attending face-to-face appointments since the onset of the cost-of-living crisis: 65.9% to 64.1% (−1.8%, *P* = 0.01). At the same time, more people are now choosing virtual appointments: 7.0% to 9.5% (+2.5%, *P* < 0.001). 23.4% of service users did not have any specific preference in the modality of care delivery before the crisis and this number stayed generally the same: 22.5% (*P* = 0.4) (Figure 1). In Germany, there was an increase in public preferences for both face-to-face and virtual appointments: 70.5% to 74.2% (+3,7%, *P* < 0.001) and 6.6% to 8.6% (+2.0%, *P* = 0.008), respectively. The number of participants who did not express any preference for appointment modality substantially decreased after the onset of the crisis: 17.9% to 12.1% (−5.8%, *P* < 0.001) (Figure 2). Italy had the highest number of respondents who preferred face-to-face medical appointments before the crisis: 80.8%, which decreased by 2.7% (*P* = 0.002). At the same time, 3.8% more people currently prefer virtual appointments with their doctors (*P* < 0.001). The number of respondents who did not express any preference for the mode of medical care delivery did not significantly change (*P* = 0.13) (Figure 3). Finally, in Sweden, an increase in preference for both face-to-face and virtual visits has been observed: +4.0% (*P* < 0.001) and +1.1% (*P* = 0.07), respectively and the number of people with no preference has decreased since the onset of the crisis −4.6%, (*P* < 0.001) (Figure 4).

ns: not significant, *: *P* < 0.05, **: *P* < 0.01, ***: *P* < 0.001; all calculations have been performed based on a full sample of participants (*n* = 8152).

ns: not significant, *: *P* < 0.05, **: *P* < 0.01, ***: *P* < 0.001; all calculations have been performed based on a full sample of participants (*n* = 8152).

ns: not significant, *: *P* < 0.05, **: *P* < 0.01, ***: *P* < 0.001; all calculations have been performed based on a full sample of participants (*n* = 8152).

ns: not significant, *: *P* < 0.05, **: *P* < 0.01, ***: *P* < 0.001; all calculations have been performed based on a full sample of participants (*n* = 8152).

Overall, there has been a tangible increase in public preference for virtual care compared to pre-crisis times in all countries. At the same time, face-to-face appointments remain the most favoured mode of healthcare delivery.

### Factors that influenced public preferences for healthcare delivery

#### Baseline preference towards virtual care

In this study, age and urban residency had the most significant impact on participants’ choices regarding virtual care before the cost-of-living crisis. Older people were less likely to choose virtual care than younger people (OR 0.966, 95% CI 0.961–0.972, *P* < 0.001). At the same time, urban residents more often preferred virtual care (OR 1.28, 95% CI 1.05–1.56, *P* = 0.01). These factors remained statistically significant in the multivariate analysis (Table 4).

**Table 4.** Factors that influence baseline preference (i.e. before COL) towards virtual care.

	Univariate		Multivariate	
	OR (95%CI)	*P*-value	OR (95% CI)	*P*-value
**Location**				
**Urban**	1.32 (1.08–1.60)	0.006	**1.28 (1.05–1.56)**	0.**01**
Rural/mixed (reference)				
**Income**				
Lower tercile	1.2 (0.95–1.51)	0.13	^a^	
Upper tercile	1.21 (0.95–1.54)	0.12	^a^	
Middle tercile (reference)				
**Gender**				
Male (reference)				
Female	0.93 (0.77–1.11)	0.44	^a^	
**Age**	0.966 (0.961–0.972)	<0.001	**0.966 (0.961–0.972)**	**<**0.**001**

^a^ This variable was not included in the multivariate analysis due to its lack of significance in the univariate analysis.

#### Factors that influenced change in public preferences

Approximately 17% (*n* = 1090) of the participants changed their preference for healthcare delivery after the onset of the crisis. In this group, the lower income quantile was associated with a change of preference towards face-to-face care (OR 1.34, 95% CI 1.12–1.62, *P* = 0.002) and older adults were less likely to change their preference either towards virtual (OR 0.973, 95% CI 0.967–0.979, *P* < 0.001), or face-to-face care (OR 0.994, 95% CI 0.988–0.999, *P* = 0.03) (Tables 5 and 6).

**Table 5.** Factors that influenced the change in public preference towards virtual care.

	Univariate		Multivariate	
	OR (95% CI)	*P*-value	OR (95% CI)	*P*-value
**Location**				
Urban	1.01 (0.83–1.24)	0.89	^a^	
Rural/mixed (reference)				
**Income**				
Lower tercile	1.32 (1.03–1.69)	0.03	^b^	
Upper tercile	1.20 (0.92–1.56)	0.18	^a^	
Middle tercile (reference)				
**Gender**				
Male (reference)				
Female	1.08 (0.89–1.31)	0.45	^a^	
**Age**	0.973 (0.967–0.979)	<0.001	**0.973 (0.967–0.979)**	**<0.001**

^a^ This variable was not included in the multivariate analysis due to its lack of significance in the univariate analysis.

^b^ This variable was not significant in the multivariate analysis.

**Table 6.** Factors that influenced the change in public preference towards face-to-face care.

	Univariate		Multivariate	
	OR	*P*-value	OR	*P*-value
**Location**				
Urban	0.88 (0.75–1.04)	0.15	^a^	
Rural/mixed (reference)				
**Income**				
Lower tercile	1.29 (1.05–1.60)	0.02	**1.34 (1.12–1.62)**	**0**.**002**
Upper tercile	0.91 (0.72–1.15)	0.42	^a^	
Middle tercile (reference)				
**Gender**				
Male (reference)				
Female	1.07 (0.91–1.26)	0.38	^a^	
**Age**	0.993 (0.988–0.998)	0.009	**0.994 (0.988–0.999)**	**0**.**03**

^a^ This variable was not included in the multivariate analysis due to its lack of significance in the univariate analysis.

## Discussion

### Key findings

Since the onset of the cost-of-living crisis, a statistically significant increase in preference for virtual care has been observed in the United Kingdom, Germany and Italy. Before the crisis, people who preferred virtual care were mainly younger and from an urban background.

Approximately 17% of included participants changed their preference for the modality of care after the onset of the cost-of-living crisis. Among them, younger people were more likely to switch to virtual care, while change to face-to-face was associated with younger age and lower income. Older adults were less likely to change their preference for either of the modalities.

### Comparison with existing literature

The cross-country variations in public preferences for the modality of healthcare delivery can be attributed to differences in health systems, policy contexts and societal factors. For instance, in Germany, the mean waiting times for primary care appointments are four days, even shorter for privately insured patients.^16^ At the same time, in the United Kingdom, patients sometimes need to wait up to two weeks to see their GP.^17^ Due to possible or anticipated long waiting times for an in-person appointment, some people might opt for alternative ways to speak with the doctor, such as available online services.

Despite the abovementioned differences between countries, a trend towards virtual consultations has been observed since the start of the cost-of-living crisis. Generally, this aligns with a significant body of other evidence which, although it did not specifically look at the use of virtual consultations during the cost-of-living crisis, reported a stark increase in the prevalence of telemedicine during the pandemic.^18–21^

Primary care physicians have been advised to return to offering in-person appointments without prior triage following the downgrading of the pandemic.^22^ Nevertheless, our study shows that virtual care not only remains an indispensable part of medical care but seems to have increased in popularity among service users in light of the ongoing cost-of-living crisis. It can be assumed that the benefits of virtual care, such as reduced travel costs, time efficiency, better work or childcare flexibility, are particularly relevant for patients and may encourage them to choose virtual care over face-to-face.^23^ At the same time, even in countries with universal health coverage where medical care is accessible to everyone, digital health, unfortunately, is not.

Socio-economic disparities in the use of digital health have been acknowledged in a significant body of existing evidence. In their cohort study, Darrat et al. found that increasing age and being in the lowest household income quartile were associated with lower chances of completing a virtual care visit, which corresponds with the results we obtained in this study.^24^ Similar results have been reported by Agarwal et al., who conducted a cross-sectional survey at primary care practices in Canada and found that people from poorer households were less comfortable using virtual care and had a stronger preference for in-person appointments.^25^

Similar to a growing body of other literature, our findings indicate a strong link between socio-demographic determinants and patients’ use of digital care and highlight the existing inequalities in this matter. As virtual care has become an integral part of today's health services, considerable efforts should be made to support disadvantaged groups such as older adults, people from rural areas and lower-income households. Further research is needed to explore the reasons for lower preference towards virtual care in these patient categories, as this will help develop patient-centred support strategies in using digital health.

### Strengths, limitations and future research

YouGov utilises active sampling and matching of the respondents to ensure that the survey results are representative of each population.^15^ Therefore, the effects of selection bias in this study are limited, however, the effect of non-responsiveness may still affect the data. Points, which can later be redeemed for cash or gift cards, are awarded for questionnaire completion; therefore, non-completion rates may be lower than for non-incentivised surveys. As a result, the data in this study may not be significantly influenced by non-responsiveness.^15^ By performing a multivariate analysis of the factors affecting the preferences of respondents, further areas of research are identified to allow exploration of the rationale for this and to allow the creation of interventions to address them. This research, however, is subject to some limitations. Single mode of survey administration (electronic) could have contributed to certain selection bias, as the study predominantly captured opinions of people who are, to some extent, familiar with the digital technology (i.e. using the internet and computers) and could have missed the perspectives of those who rarely or never use digital technology. The questionnaire design required participants to assess their preferences for the mode of healthcare delivery retrospectively. While this was unavoidable in this study, future research could utilise a concurrent quantitative approach to record the utilisation of virtual and face-to-face consultations from 2021 in each country and assess the degree to which this aligned with responses. Variables such as average wait for face-to-face and virtual appointments, previous experience with virtual care and consultation modalities available to patients both pre-2021 and presently were not captured in this study. It is, therefore, not possible to understand the rationale behind participants’ preferences in this study. Finally, due to the heterogeneity of ethnicity data, we could not include this variable in the regression analysis, which could have provided additional insights into the matter.

### Implications for policy

With the 2023 government mandate for England's National Health Service explicitly citing the increased utilisation of ‘data and technology to drive elective recovery’, the results of this paper should be considered at all levels, from national health to local policy development.^26^ Our findings show a growing demand for virtual consultations, particularly by younger people. However, this was not mirrored by those who consume the most healthcare resources (i.e. older people and those from lower-income groups).^27,28^

Scaling up digital healthcare will, therefore, prove a challenging equilibrium to strike to ensure that the wants and needs of the younger population are met while not alienating the older population and those more deprived of their healthcare providers. Policymakers must be acutely aware of this and consider strategies to ensure equitable access to virtual care across all stages of its conception and implementation. Such strategies could include digital health literacy training, creating comprehensible guidance materials, and community support initiatives. Our results also indicate that participants from rural communities preferred face-to-face appointments rather than virtual consultations. While our study did not explore the rationale for this, future work may have implications for the industry to ensure equitable coverage of internet networks and digital health hubs to improve access to virtual services for rural communities.

## Conclusion

Public preference for virtual care has significantly increased after the onset of the cost-of-living crisis, even as the COVID-19 pandemic has receded. Subjects that prefer virtual consultations over in-person visits are younger, while older adults and people with lower-than-average income are most likely to choose face-to-face care. The rationale behind patients’ preferences should be investigated to ensure all patients can access care in their preferred modality. New policies should be developed to provide equitable access to digital care for patients who could benefit from it.
